# The Amsterdam Wrist Rules: how much money can they save?

**DOI:** 10.1007/s10198-020-01168-x

**Published:** 2020-03-17

**Authors:** Marjolein A. M. Mulders, Monique M. J. Walenkamp, Nico L. Sosef, Frank Ouwehand, Romuald van Velde, J. Carel Goslings, Niels W. L. Schep

**Affiliations:** 1 Trauma Unit, Department of Surgery, Amsterdam UMC, Academic Medical Center, University of Amsterdam, P.O. Box 22660, 1100 DD Amsterdam, The Netherlands; 2 Department of Surgery, Spaarne Gasthuis, P.O. Box 770, 2130 AT Hoofddorp, The Netherlands; 3 Emergency Department, Amsterdam UMC, Academic Medical Center, University of Amsterdam, P.O. Box 22660, 1100 DD Amsterdam, The Netherlands; 4 Department of Surgery, Tergooi Hospitals, P.O. Box 10016, 1201 DA Hilversum, The Netherlands; 5 Department of Surgery, Onze Lieve Vrouwe Gasthuis, P.O. Box 95500, 1090 HM Amsterdam, The Netherlands; 6 Department of Trauma and Hand Surgery, Maasstad Hospital, P.O. Box 9100, 3007 AC Rotterdam, The Netherlands

**Keywords:** Distal radius, Fracture, Trauma, Decision rule, Radiograph, Cost analysis, I100

## Abstract

**Purpose:**

To allow physicians to be more selective in their request for a radiograph of the wrist and to potentially reduce costs, the Amsterdam Wrist Rules (AWR) have been developed, externally validated, and recently also implemented. The aim of this study was to conduct an incremental cost analysis and budget impact analysis of the implementation of the AWR at the emergency department (ED) in the Netherlands.

**Methods:**

A cost-minimisation analysis to determine the expected cost savings for implementation of the Amsterdam Wrist Rules. The incremental difference in costs before and after implementation of the AWR was based on the reduction in costs for radiographs, the cost savings due to reduction of ED consultation times and the costs of a re-evaluation appointment by a physician.

**Results:**

In the Netherlands, implementation of the AWR could potentially result in 6% cost savings per patient with a wrist injury. In addition, implementation of the AWR resulted in €203,510 cost savings annually nationwide. In the sensitivity analysis, an increase in physician compliance to 100% substantially increased the potential total amount of annual cost savings to €610,248, which is 6% of total costs before implementation. Variation in time spent at the ED, a decrease and increase in costs and patients presenting annually at the ED did not change the cost savings substantially.

**Conclusion:**

Implementation of the AWR has been shown to reduce direct and indirect costs and can, therefore, result in considerable savings of healthcare consumption and expenditure.

**Electronic supplementary material:**

The online version of this article (10.1007/s10198-020-01168-x) contains supplementary material, which is available to authorized users.

## Introduction

A trauma of the wrist is a common reason for a patient to present at the emergency department (ED). However, only half of the patients who present at the ED have sustained a fracture of the wrist [1, 2]. Nonetheless, almost all patients undergo radiographs of the wrist. Even though the costs per radiograph are limited, due to the high incidence of wrist trauma the cumulative costs can be substantial. The estimated total healthcare costs for wrist fractures in the Netherlands are 83 million euro per year, making them the most expensive injuries of the upper extremity [3, 4].

To allow physicians to be more selective in their request for a radiograph, the Amsterdam Wrist Rules (AWR) have been developed, externally validated [1], and recently also implemented [5]. Based on the age of the patient and several clinical variables present during physical examination, the AWR provide a recommendation to request a radiograph of the wrist or not in patients suspected of a distal radius fracture. After implementation of the AWR, an absolute reduction of 15.3% in wrist radiographs was found, without missing any clinically relevant fractures. Moreover, due to the AWR non-fracture patients without a wrist radiography spent 34 min less at the ED compared with non-fracture patients who had a wrist radiograph. This reduction in radiographs requested and time spent at the ED could potentially result in cost savings.

Therefore, the aim of this study was to conduct an incremental cost analysis for the implementation of the AWR at the ED in the Netherlands. Secondary, we aimed to conduct a budget impact analysis to estimate the total impact on the healthcare budget in the Netherlands.

## Methods

In this study, we used a diagnostic technology evaluation to determine the expected cost savings for the implementation of the Amsterdam Wrist Rules [6]. The total costs of treatment for two cohorts of patients were calculated based on their use of resources. Data were retrieved from the recent implementation study of the Amsterdam Wrist Rules [5]. This before and after study, compared 402 patients in which the AWR were implemented (after group), with a prospectively collected historical reference group of 859 patients in which the AWR were not yet implemented (before group). The estimates of fracture prevalence and the reduction in radiographs were based on this recent implementation study. Patients in the after group were included using a smartphone application developed for use of the AWR. All patients that did not receive a radiograph of the wrist were telephoned after 1 week to determine if a fracture was missed and if any subsequent physician appointments or radiographs were performed.

The baseline characteristics age, gender and percentage of distal radius fractures of the before and after group were comparable (Table 1). The absolute reduction in wrist radiographs was 15.3% (99.4% versus 84.1%; *p* < 0.001); before the implementation of the AWR, in 0.6% of patients no radiograph was requested, compared to 15.9% after implementation. 36% of the physicians adhered to the recommendation. Non-fracture patients without a wrist radiography due to the AWR spent 34 min less at the ED compared with non-fracture patients who had a wrist radiograph (*p* = 0.015). This comprises a 29% reduction in time spent at the ED compared with the period before implementation of the AWR [5].

**Table 1:** Baseline characteristics before and after implementation of the AWR

	Before implementation AWR	After implementation AWR	*p* value
	*N* = 859	*N* = 402	
Age [median (IQR)]	50 (31–63)	51 (32–67)	0.294
Female (%)	60.5	60.7	0.957
Distal radius fractures (%)	43	44	0.814
Wrist radiographs (%)	99.4	84.1	< 0.001

*N* number, *IQR* interquartile range

One patient had a subsequent outpatient clinic appointment and wrist radiograph because she still had complaints when she was phoned after 1 week. She received a removable splint for 4 weeks for a clinically irrelevant fracture.

### Cost analysis

Considering that the AWR should not result in differences in health outcomes, the economic evaluation was set up as a cost-minimisation analysis and addressed direct medical costs (i.e. use of radiographs, consultation at the ED and other healthcare providers, decrease in the length of the ED consultation) and nonmedical costs (i.e. travel expenses to the ED and for additional hospital appointments). Costs for each consultation at the ED (both with and without a radiograph) were estimated and any additional consultations at the ED or other healthcare providers were also included. It was assumed that no missed fracture would remain undetected indefinitely and that possible fractures missed at the ED due to the AWR would not have been missed if the patient had undergone radiography. Therefore, we considered additional radiographs and treatment related to missed fractures as delayed costs and not as additional costs. The subsequent outpatient clinic appointment and travel expenses to the outpatient clinic were considered as the only additional medical cost. Cost savings realized by shorter consultation times at the ED were considered as well since this time could be spent for another patient resulting in better use of resources. Since we did not expect that a delayed diagnosis would influence the absence at work, we did not take into account the productivity loss of patients. Moreover, since the variables of the AWR are part of the physical examination, we considered the additional time of completing the mobile application negligible.

Charges (in Euros) of radiographs of the wrist, ED consultations and outpatient clinic appointments, and travel expenses were obtained from the Dutch costs manual 2016 and extrapolated to 2015 using the consumer price index [7]. Travel expenses were based on a charge of 19 euro cents per kilometre, with an average travel distance of seven kilometres. An additional three euros was added for the parking fee. Values of all costs used in this analysis are displayed in Table 2. Since the AWR had a 100% sensitivity on detecting clinically relevant fractures, the baseline analysis focused on the incremental cost difference between standard practice and application of the AWR. A sensitivity analysis was performed on increasing the physician compliance, the time spent at the ED, and increasing and decreasing the costs for a radiograph, the consultation at the ED, and additional outpatient clinic appointment.

**Table 2:** Costs per item

ED consultation	260.55
ED consultation with reduction in length of stay at ED	185.51
Outpatient clinic appointment	91.55
Radiograph of wrist	47.02
Travel expenses	4.36

All values are displayed in Euros

*ED* emergency department

With these assumptions, the incremental difference in costs before and after implementation of the AWR was based on the reduction in costs for wrist radiographs, the cost savings due to reduction of ED consultation times and the costs of a re-evaluation appointment by a physician.

### Budget impact analysis

In a budget impact analysis, the study results of the cost analysis were extrapolated to the national level to estimate the total impact on the healthcare budget per annum for the Netherlands in terms of health benefits. The budget impact analysis was based on an estimated number of 34,500 adult patients with a trauma of the wrist presenting annually at the ED. This number was based on the Dutch Injury Surveillance System (LIS) [8]. This national registry registers all trauma patients who present at the ED of a representative sample of hospitals in the Netherlands.

A sensitivity analysis was performed on increasing physician compliance, and time spent at the ED. Moreover, a sensitivity analysis was performed on increasing and decreasing the costs for a radiograph and additional outpatient clinic appointment, and the number of patients presenting at the ED.

## Results

### Cost analysis

Table 3 shows the (incremental) cost savings in the total cohort of 402 patients. Considering an absolute reduction of 15.3% in wrist radiographs (99.4% requested radiographs before implementation versus 84.1% after implementation), the total cost savings after implementation of the AWR were €2420 for the baseline analysis. This is 2% savings per patient with a wrist injury after implementation of the AWR.

**Table 3:** Base case analysis for cost savings

	Before implementation		After implementation	
	Number of patients	Total costs	Number of patients	Total costs
ED consultation	402	104,222	379	98,750
ED consultation without wrist radiograph	2	371	23	4267
Outpatient clinic appointment	0	–	1	92
Radiograph of wrist	400	18,808	380	17,868
Travel expenses	402	1751	403	1755
Total base case		125,152		122,732

All values are displayed in Euros

*ED* emergency department

**Fig. 1:**
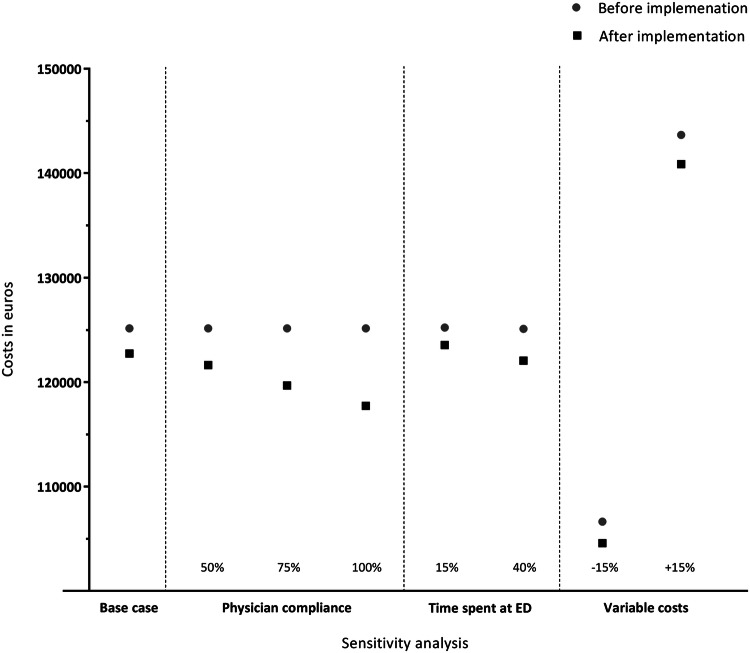
Cost savings after implementation of the AWR, including sensitivity analysis

In the sensitivity analysis, when considering the reduction in radiographs due to an increase in physician compliance of 50%, 75% and 100%, a total amount of €3519, €5472 and €7425, respectively, could potentially be saved. When considering a 100% physician compliance, the savings per patient tripled to 6%. If the reduction in ED length of stay would be decreased to 15%, the difference in costs decreased as well (cost savings €1665). In contrast, if the reduction in ED length of stay would be increased to 40%, the difference in costs was increased as well (cost savings €3034). The same applied if the costs for a radiograph, the consultation at the ED, and additional outpatient clinic appointment would be decreased and increased with 15% (€2057 and €2784 cost savings, respectively) (Fig. 1, Appendix 1).

### Budget impact analysis

Finally, the cost savings were translated to cost savings for the Dutch population (Table 4). In the Netherlands, an estimated number of 34,500 patients annually present at the ED due to a trauma of the wrist. Based on this number of patients, implementation of the AWR resulted in €203,510 cost savings per year.

**Table 4:** Base case analysis for budget impact analysis

	Before implementation		After implementation	
	Number of patients	Total costs	Number of patients	Total costs
ED consultation	34,293	8,935,178	32,525	8,474,519
ED consultation without wrist radiograph	207	38,401	1,975	366,391
Outpatient clinic appointment	0	–	86	7873
Radiograph of wrist	34,293	1,612,473	32,611	150,656
Travel expenses	34,500	150,281	34,586	1,533,385
Total base case		10,736,334		10,532,824

All values are displayed in euros

*ED* emergency department

In the sensitivity analysis, the potential total cost savings based on an increase in physician compliance varied between €610,247 (100% physician compliance), €451,311 (75% physician compliance) and €292,490 (50% physician compliance). The cost savings after increasing the physician compliance to 100%, is 6% of total costs before implementation. In addition, if the reduction in time spent at the ED was assumed to decrease to 15%, €139,954 was saved. In contrast, if the reduction in time spent at the ED was assumed to increase to 40%, €255,089 was saved. Moreover, a decrease of 15% in costs would result in €172,943 cost savings, and an increase of 15% would result in €234,078 cost savings.

**Fig. 2:**
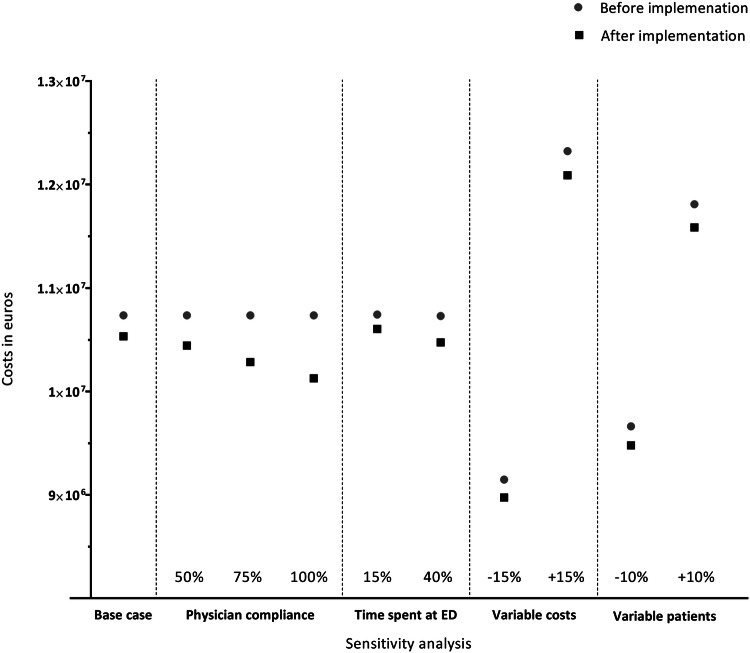
Budget impact analysis after implementation of the AWR, including sensitivity analysis

Last, keeping in mind a different estimated number of patients presenting at the ED in the Netherlands annually, the cost savings would be €183,193, based on an estimated number of 31,050 patients (10% decrease), and €223,850, based on an estimated number of 37,950 patients (10% increase) presenting at the ED annually (Fig. 2, Appendix 2).

## Discussion

In this study, the cost analysis and budget impact analysis of implementation of the AWR at the ED in the Netherlands was established under a variety of different assumptions. Based on the 402 included patients, implementation of the AWR results in €2420 cost savings. When increasing the physician compliance to 100%, implementation of the AWR could potentially result in 6% cost savings per patient with a wrist injury. Additionally, based on an estimated number of 34,500 patients annually presenting at the ED with a trauma of the wrist, implementation of the AWR could potentially reduce the costs with €203,511. Sensitivity analysis that varied the time spent at the ED, the rate of costs for a radiograph and additional outpatient clinic appointment, and the amount of patients presenting at the ED, did not change the results substantially. Yet, under each assumption of the sensitivity analysis, implementation of the AWR resulted in cost savings compared to current practice. Moreover, an increase in the physician compliance tripled the decrease in costs for both the cost analysis and the budget impact analysis to an amount of €7425 and €610,248 (both 6% of total costs before implementation), respectively.

The potential cost savings of the implementation of the AWR could be beneficial for hospitals or healthcare insurance companies. Although these savings may not result in a significant reduction in the total annual healthcare expenditures, they liberate resources that can be used elsewhere resulting in better use of resources at the ED. In contrast to the total annual healthcare expenditures of approximately 95 billion euro in the Netherlands in 2015, the reduction of radiographs, which individually costs little but which is frequently used by physicians, could account for far more in the annual growth of healthcare expenditures than do a few big technologies [9].

Since this study was conducted using a societal perspective, time spent at the ED was incorporated. Ideally, this should be calculated by analyzing the willingness to pay of these patients for time-saving activities [10]. However, we assumed that a 29% reduction in time spent at the ED after implementation of the AWR, equalled the percentage of costs saved. Loss of productivity was not taken into account since we assumed that a delayed diagnosis would not result in additional days off work. Yet, the cost for the additional outpatient clinic appointment and the delayed radiograph were taken into account. To control for potential errors in this assumption, a sensitivity analysis was performed by decreasing and increasing the length of stay at the ED and thereby the costs. Although the different sensitivity analyses did not change the results of both the cost analysis and the budget-impact analysis substantially, increasing the compliance of the physicians to 100% tripled the costs savings. We expect that, by demonstrating that the AWR can safely be used, the adherence of physicians towards the AWR will increase in the future and therefore increasing the cost savings. Moreover, despite the evidence that the AWR can safely reduce the amount of wrist radiographs requested and thereby reducing costs, physicians may still feel uncomfortable about using the AWR. This is mostly related to the concern of missing a fracture in patients who did not receive a wrist radiograph, and the possible medicolegal consequences [11]. However, if the AWR will be generally accepted as good clinical practice and endorsed by (inter)national societies, it is not very likely that implementation would lead to liability [12, 13].

Finally, radiography of the wrist was assumed to have a 100% sensitivity and specificity for identifying fractures of the distal radius, and therefore we assumed that no fractures were missed in the before group. Although the physicians’ ability to rule out a distal radius fracture is high [14], fractures of the distal radius are sometimes missed. Either because of misinterpretation of the physician or because the fracture was not visible on radiography. Therefore, it is possible that the assumption of no missed fractures in the before group was an underestimation, causing an underestimation of the cost difference.

In conclusion, the AWR have been shown to safely reduce the number of wrist radiographs requested at the ED and consequently the time spent at the ED. Moreover, after the incorporation of direct and indirect costs, the implementation of the AWR has also been shown to reduce costs. Implementation of the AWR would, therefore, result in considerable savings of healthcare expenditures, and supports the introduction of the AWR into clinical practice, from both a clinical as well as health economic point of view.

## Electronic supplementary material

Below is the link to the electronic supplementary material.

Supplementary material 1 (DOCX 12 kb)

Supplementary material 2 (DOCX 13 kb)
