# Supplementary material for: The Amsterdam Wrist Rules: how much money can they save?
Source: Eur J Health Econ. 2020 Mar 17;21(5):745–50. doi: 10.1007/s10198-020-01168-x (PMC7366574; doi:10.1007/s10198-020-01168-x)
Supplement: Supplementary file 1 — Supplementary material 1 (DOCX 12 kb) [file 10198_2020_1168_MOESM1_ESM.docx]

| ***Appendix 1. Cost savings after implementation of the AWR*** | | | |
| --- | --- | --- | --- |
|  | **Before implementation** | **After implementation** | **Cost difference** |
| **Base case analysis** | 125,152 | 122,732 | -2,420 |
| **Sensitivity analysis** |  |  |  |
| **Physician compliance**  **50% compliance**  **75% compliance**  **100% compliance** | 125,152  125,152  125,152 | 121,633  119,680  117,727 | -3,519  -5,472  -7,425 |
| **Time spent at the ED**  **15% reduction ED length of stay**  **40% reduction ED length of stay** | 125,224  125,094 | 123,559  122,060 | -1,665  -3,034 |
| **Variable cost units**  **15% decrease in costs**  **15% increase in costs** | 106,642  143,662 | 104,585  140,878 | -2,057  -2,784 |
| All values are displayed in euros. ED: emergency department | | | |
