# Supplementary material for: The Amsterdam Wrist Rules: how much money can they save?
Source: Eur J Health Econ. 2020 Mar 17;21(5):745–50. doi: 10.1007/s10198-020-01168-x (PMC7366574; doi:10.1007/s10198-020-01168-x)
Supplement: Supplementary file 2 — Supplementary material 2 (DOCX 13 kb) [file 10198_2020_1168_MOESM2_ESM.docx]

| ***Appendix 2. Budget impact analysis after implementation of the AWR*** | | | |
| --- | --- | --- | --- |
|  | **Before implementation** | **After implementation** | **Cost difference** |
| **Base case analysis** | 10,736,334 | 10,532,824 | -203,510 |
| **Sensitivity analysis** |  |  |  |
| **Physician compliance**  **50% compliance**  **75% compliance**  **100% compliance** | 10,736,334  10,736,334  10,736,334 | 10,443,844  10,285,023  10,126,087 | -292,490  -451,311  -610,247 |
| **Time spent at the ED**  **15% reduction ED length of stay**  **40% reduction ED length of stay** | 10,743,777  10,730,293 | 10,603,823  10,475,204 | -139,954  -255,089 |
| **Variable cost units**  **15% decrease in costs**  **15% increase in costs** | 9,148,483  12,323,877 | 8,975,540  12,089,799 | -172,943  -234,078 |
| **Patients presenting at ED**  **10% decrease in patients**  **10% increase in patients** | 9,662,738  11,809,931 | 9,479,545  11,586,081 | -183,193  -223,850 |
| All values are displayed in euros. ED: emergency department | | | |
